# Improving Imperfect Data from Health Management Information Systems in Africa Using Space–Time Geostatistics

**DOI:** 10.1371/journal.pmed.0030271

**Published:** 2006-06-06

**Authors:** Peter W Gething, Abdisalan M Noor, Priscilla W Gikandi, Esther A. A Ogara, Simon I Hay, Mark S Nixon, Robert W Snow, Peter M Atkinson

**Affiliations:** **1**School of Geography, University of Southampton, Southampton, United Kingdom,; **2**School of Electronics and Computer Science, University of Southampton, Southampton, United Kingdom,; **3**Malaria Public Health and Epidemiology Group, Centre for Geographic Medicine, Kenya Medical Research Institute/Wellcome Trust Collaborative Programme, Nairobi, Kenya,; **4**Ministry of Health, Division of Health Management Information System, Nairobi, Kenya,; **5**Spatial Ecology and Epidemiology Group, Department of Zoology, University of Oxford, Oxford, United Kingdom,; **6**Centre for Tropical Medicine, University of Oxford, John Radcliffe Hospital, Oxford, United Kingdom; University of QueenslandAustralia

## Abstract

**Background:**

Reliable and timely information on disease-specific treatment burdens within a health system is critical for the planning and monitoring of service provision. Health management information systems (HMIS) exist to address this need at national scales across Africa but are failing to deliver adequate data because of widespread underreporting by health facilities. Faced with this inadequacy, vital public health decisions often rely on crudely adjusted regional and national estimates of treatment burdens.

**Methods and Findings:**

This study has taken the example of presumed malaria in outpatients within the largely incomplete Kenyan HMIS database and has defined a geostatistical modelling framework that can predict values for all data that are missing through space and time. The resulting complete set can then be used to define treatment burdens for presumed malaria at any level of spatial and temporal aggregation. Validation of the model has shown that these burdens are quantified to an acceptable level of accuracy at the district, provincial, and national scale.

**Conclusions:**

The modelling framework presented here provides, to our knowledge for the first time, reliable information from imperfect HMIS data to support evidence-based decision-making at national and sub-national levels.

## Abstract 2

When adopted to health systems reporting, space-time kriging can estimate large numbers of missing data to acceptable levels of accuracy, and thus allow evidence-based decision making and allocation of resources.

## Introduction

Public health decision-makers require accurate and timely information on disease-specific treatment burdens within a health system to monitor and plan resource needs [ 1– 4]. A basic requirement is reliable national and sub-national data detailing the number of treatment events for a given disease or condition occurring at health facilities each month or year. In most African settings, this requirement is addressed with a health management information system (HMIS) that coordinates the routine acquisition of treatment records from health facilities and the transfer, compilation, and analysis of these data through district, regional, and national levels.

A perfect HMIS requires all health facilities to report promptly in all months, allowing a comprehensive quantification of treatment events through time and space across the health system. The reality of HMIS in Africa and elsewhere stands in marked contrast to this ideal [ 5– 9]. Typically, many facilities never report, or report only intermittently, resulting in spatially and temporally incomplete national data [ 10– 13]. Following several decades of donor investment in HMIS across Africa, the incomplete nature of routine national reporting has shown little improvement [ 3, 14].

Faced with poor data coverage, national treatment burdens are often estimated using rudimentary methods to account for missing values. The objective of this paper is to present a geostatistical model that predicts missing data in order to provide more reliable estimates of national outpatient treatment burdens with known accuracy. The model has been developed and tested using the example of presumed malaria cases in the Kenyan government's formal health sector.

## Methods

### The Kenyan HMIS Dataset

Data were obtained from the Department of Health Management Information Systems of the Kenyan Ministry of Health. These data consisted of monthly records of diagnoses made at outpatient departments of health facilities across Kenya over an 84-mo period (January 1996–December 2002). Each record included the total number of all-cause diagnoses made at a given facility during a given month. An additional 11 diagnostic codes were available for each monthly record per facility. We selected malaria as the diagnostic code for model development for a number of reasons: (a) it accounted for over a third of all diagnoses made during the period of observation; (b) malaria is a disease that demands accurate quantification for health system planning in the light of increased donor assistance [ 9], particularly in the era when new expensive therapeutics are being adopted [ 9, 15]; and (c) malaria exhibits considerable spatial [ 16, 17] and temporal [ 18, 19] heterogeneity across Kenya. The records available within the routine HMIS data were not structured by age or sex, nor were they distinguished as initial or follow-up visits, and diagnoses were generally not slide-confirmed. The data, therefore, represent total cases (TC) or presumed malaria cases (MC) seen as outpatients each month at health facilities identified by a unique facility code.

Data for each facility were matched to an independent database indicating the longitude and latitude of formal government, mission, and private health facilities nationwide. Details of how this spatial database was constructed are provided elsewhere [ 20] and were updated in 2005 [ 21]. In this paper, we focus on the government providers of routine outpatient care in order to assess treatment burdens within this sector, although the techniques presented can be extended to include georeferenced facilities within any given sector. Government health facilities at the district level are structured according to the levels of service they provide, with the most sophisticated being the general hospitals supporting a network of health centres that in turn act as referral points from dispensaries at the periphery.

### Space–Time Geostatistics

A straightforward technique for predicting national MC totals using incomplete data is to scale up the tally of cases from available records in proportion to the number of missing data. This simplistic approach neglects any heterogeneity in the pattern of MC through space and time across the country. A more sophisticated approach is to predict each missing record individually from existing data. In the presence of spatial and temporal heterogeneity in MC, it is intuitive to allow data that are proximate to the record being predicted to have more influence on its prediction than those that are distant. In a traditional geostatistical approach [ 22, 23], the nature of spatial heterogeneity in the variable of interest is modelled explicitly using a variogram function that relates dissimilarity (quantified using semivariance) to spatial separation (termed lag). This function is then used to determine optimal data weightings in an interpolation exercise such as ordinary kriging, which predicts missing values using a weighted linear average of proximate data. Space–time kriging (STK) is an extension of ordinary kriging that considers simultaneously spatial and temporal heterogeneity and can provide more accurate predictions when the variable of interest is distributed through time as well as a space [ 24– 27]. The one-dimensional spatial variogram function is replaced with a two-dimensional space–time variogram, and the kriging algorithms are adapted to make predictions using spatially and temporally proximate data ( Protocol S1).

### Model Development

We used STK to predict MC values at facilities where monthly records were missing. The accuracy of geostatistical predictions is greatly influenced by the amount of spatial correlation present in the variable of interest, that is, the extent to which values vary smoothly through space. The spatial structure of MC values at different facilities is confounded by facility-specific factors such as their type, catchment population size, and utilisation. These factors are not constrained spatially in the same way as malaria risks and may vary widely between facilities, regardless of their spatial proximity. To increase the predictive accuracy of STK it was necessary to increase the spatial correlation of the predicted variable by standardising MC by these facility-specific factors. This standardisation was achieved by dividing each monthly MC value by the mean monthly TC (MMTC) at each facility. MMTC was used as a proxy measure of facility catchment populations, reflecting broad utilization rates driven by the facility type and catchment population densities.

**Figure 1:**
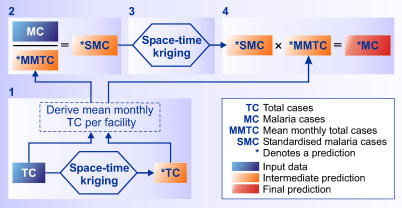
Schematic Diagram of the Modelling Framework Four stages were used to predict the count of outpatients treated for malaria (MC) for each facility-month with missing data: (1) MMTC was estimated for each facility using both existing and predicted values of TC; (2) existing MC data at each facility were standardised by the corresponding MMTC value to create SMC values; (3) STK was used to predict all missing values of SMC; and (4) MMTC values were used to back-transform the predicted SMC values in order to obtain final predictions of MC.

The modelling framework therefore consisted of several components ( Figure 1). A completed set of TC values was required for each facility (i.e., 84 continuous months) in order to estimate MMTC. This set was provided by a separate STK procedure that predicted missing TC values, *TC (where the asterisk denotes a prediction), using the existing data. The mean of the combined set of TC data and *TC predictions for each facility, *MMTC, was then calculated. *MMTC was considered a more reliable proxy of catchment population than individual monthly TC values, representing a 7-y average less susceptible to both prediction bias and short-term fluctuations in utilisation. The monthly MC data were then standardised by dividing each by the corresponding *MMTC value to estimate a new variable, standardised MC (SMC). This new variable displayed a greater amount of spatial correlation than the raw MC data. SMC data were then used in a second STK exercise to predict *SMC at all missing points. These predictions were then back-transformed to *MC by multiplication by the relevant *MMTC value. Details of the methodological steps involved in the STK exercises to predict *TC and *SMC are detailed in Protocol S1.

The above modelling framework resulted in predictions of MC at all facilities and for all months for which data were missing. In combination with the original data, this set represented a complete picture of the treatment burden for presumed malaria at all facilities for all months. This set could be aggregated to provide treatment burdens at any spatial level from the individual facility through to the district, provincial, and national levels for the 7-y period. Further, averaging could be applied to estimate values for any month or year in the set.

### Model Testing

A validation procedure was carried out to test the performance of the model in terms of the accuracy of predictions of MC at different levels of spatial and temporal aggregation. A test set of 6,349 monthly records (representing a 10% sample) was selected from the full dataset using a stratified random sampling that ensured representative proportions of each facility type. The test set was removed from the database, and the STK modelling procedure was repeated in its entirety using the remaining 90% of data to predict MC values for the test set. The resulting predictions were then compared to the reference values to provide a set of known prediction errors that could be considered a sample of the (unknown) errors of the main prediction exercise.

The total prediction error for the test set was calculated, along with the mean and standard deviation error nationwide at the level of individual facility-months. A series of subsets was then created from the test set by aggregating records together over space–time units (district-months, district-years, province-months, province-years, and so on), and the magnitude of errors was compared between subsets. The variance of these errors was found to decrease in inverse proportion to the number of records aggregated together in each subset ( Figure S1). This relationship was then used, along with the sample errors, to estimate the total prediction error and associated variance in each space–time unit. Monte Carlo simulation was used to estimate the combined distribution of total prediction errors for all space–time units in each aggregation level. This procedure resulted in, for example, estimates of the range (expressed as a 95% confidence interval) of percentage errors that could be expected for predictions of total MC for all facilities in a district over a month, all facilities in a province over a year, and so on.

## Results

### Data Coverage

A total of 2,165 government facilities were identified through consultation with district health management teams and other service providers ([ 20, 21]; A. M. Noor and P. W. Gikandi, unpublished data). It was possible to generate a longitude and latitude from various sources for over 92% of these facilities [ 21]. These included 129 hospitals, 474 health centres, and 1,399 dispensaries ( Table 1). The importance of establishing a comprehensive database was demonstrated by the identification in the above exercise of an additional 400 government facilities that were not included in the central HMIS database. A total of 163 facilities were included in this study that could not be georeferenced. Missing MC values for these facilities were estimated using the local district mean for that month.

**Table 1:**
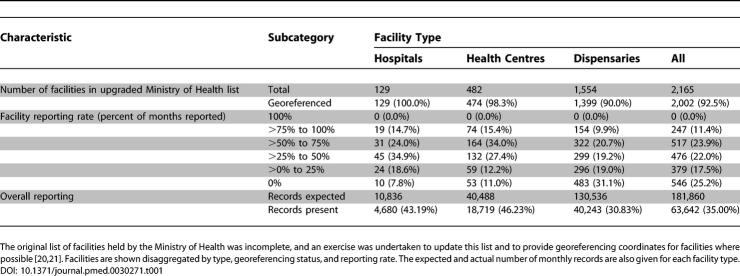
Summary of Government Health Facilities in Kenya and Their Reporting Behaviour during the 84-mo Study Period January 1996 to December 2002

### Reporting Rate

**Figure 2:**
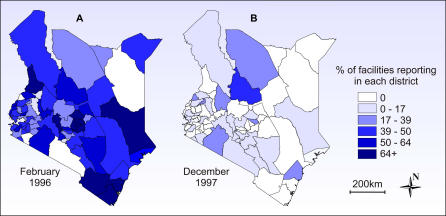
Percentage of Government Health Facilities in Each Kenyan District (Fourth Level Administrative Unit) Submitting a Monthly Outpatient Morbidity Report to the HMIS The 2 mo shown are (A) the most complete (February 1996) and (B) the least complete (December 1997) during the 84-mo study period January 1996–December 2002.

Underreporting was found to be widespread, although there was considerable variation spatially and temporally ( Figure 2) and between facility types ( Table 1). No facilities reported in all 84 mo, whilst 546 facilities (25%) did not report in any month. A complete 84-mo dataset for each of the 2,165 facilities would consist of 181,860 facility-months. There were 63,642 records, representing an overall reporting rate of 35%. The overall reporting rate varied both within and between years, with a minimum of 6% in December 1997 (this coincided with a nationwide industrial dispute by nurses) and a maximum of 44% in February 1996. The reporting rate also displayed a seasonal pattern, with generally more facilities reporting during the first three quarters of each year (36%) than in the last quarter (31%).

A total of 18.67 million cases of presumed malaria were reported, with a mean of 293.4 cases per facility-month. The totals (means) were 3.36 million (716.9) for hospitals, 6.05 million (323.4) for health centres, and 9.26 million (230.2) for dispensaries.

### Prediction of Treatment Burdens

**Figure 3:**
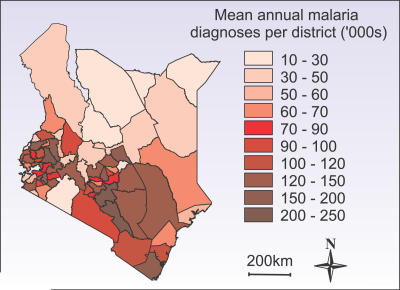
Number of Outpatients Treated for Malaria at Government Facilities Predicted mean annual totals for each district for the period 1996–2002. Values represent the combined sum of existing and predicted values.

The mean annual total of presumed malaria cases (i.e., the combined total of data plus predictions) at all government facilities between 1996 and 2002 was 6.79 million cases, with a mean of 261.5 cases per facility-month ( Table 2). The corresponding values for each facility type were 1.11 million for hospitals, 1.74 million for health centres, and 3.95 million for dispensaries, with means of 716.0, 300.3, and 211.8 cases per facility-month, respectively. Mean annual totals for each district ( Figure 3) displayed a pattern of spatial heterogeneity that corresponded broadly to a combination of malaria ecology [ 17, 28], population distribution [ 29], and facility locations [ 20].

**Table 2:**
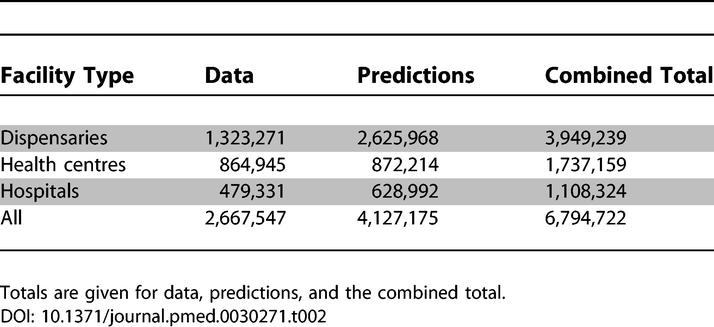
Predicted Mean Annual Counts of Outpatients Treated for Malaria at All Kenyan Government Hospitals, Health Centres, and Dispensaries for the Period 1996–2002

### Model Testing

Comparison of data with predictions for 6,349 randomly selected MC data points in the test set yielded mean prediction errors for hospitals, health centres, and dispensaries of 58.2, −8.8, and −4.7 cases per facility-month. The true and predicted sums of the entire national test set were 1,899,234 and 1,891,136, respectively, representing an overall prediction error of −0.4%.

The predictive accuracy of the model increased as predictions were made over larger aggregated space–time units ( Table 3). It was estimated that 95% of MC totals for district-months would be predicted to within 35.3% of the true value and that three-quarters would be predicted to within 15.1%. The equivalent errors for predictions of annual totals at the provincial level were 12.2% and 5.5% and at the national level were −1.3% and −0.9%.

**Table 3:**
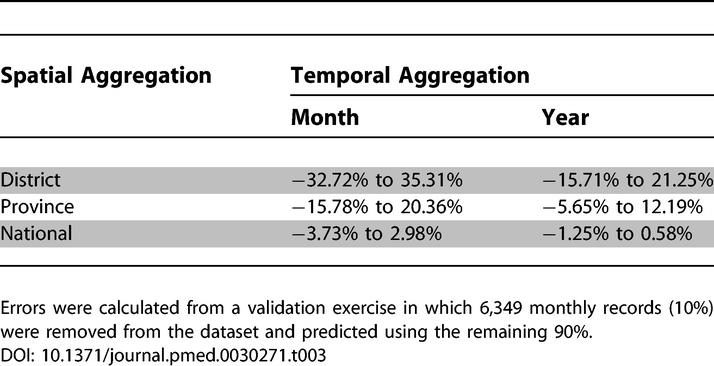
Expected Percentage Errors (95% Confidence Intervals) in Predictions of Total Outpatients Treated for Malaria over Different Levels of Spatial and Temporal Aggregation

## Discussion

Between 1996 and 2002 the Kenyan HMIS contained only 35% of the expected monthly records from government clinics providing outpatient care nationwide. This seriously limits the direct use of these data for planning health service needs, including staffing and disease-specific commodities such as anti-malarial drugs. Inadequate spatial and temporal coverage of information is compounded by a lack of information on precisely where service providers are located: only 82% of government health facilities were included in the national HMIS database. We have recently upgraded the Ministry of Health's service provider lists and have provided spatial coordinates for each health facility, and in this paper we provide a geostatistical model to improve the interpretation of incomplete data of presumed malaria cases reported to the centralised national HMIS database.

Our model accurately predicts national annual treatment burdens for presumed outpatient malaria within the government sector with an estimated margin of error of 1.3% and a predicted average of 6.8 million cases per annum over the period of observation. This demonstrates a tangible improvement over the more traditional approach of simply multiplying nationally available data by a proportion of underreporting, which results in a crude estimate of 7.6 million cases. The incidence of malaria and the proportion of individuals with the illness who seek treatment have large spatial and temporal heterogeneity, and failing to account for this heterogeneity leads inevitably to a distortion in estimates of national treatment burden. STK is a method used in atmospheric [ 30, 31] and earth sciences [ 24, 32] that we have adapted for use in our models. It is likely to provide a more precise estimation of national treatment burdens for presumed malaria at outpatient clinics, consequently allowing a more realistic approximation of treatment requirements, including new expensive anti-malarials, in this sector.

One prerequisite for STK that might limit wider application outside Kenya is that a ministry of health must have a spatially referenced map of its service providers. In Kenya, this has been made possible by the development of a geographic information system, which is applied in this paper—to our knowledge for the first time in Africa—to national HMIS data. Rather than thinking of this as a limitation to the generalisability of our approach outside Kenya, we would argue that knowing where service providers are located is a must for any health planning agency and that geographic information system frameworks for health services should be developed everywhere.

The predictive power of the proposed model decreases as predictions are required at finer spatial and temporal resolutions. Although under- and overpredictions tended to balance out when areas are aggregated, errors at individual facilities were substantial in places. Thus, different models with additional parameters, including facility drainage, facility characteristics, and competition between facilities, are likely to be required to estimate incomplete data at this level [ 33– 35]. Nevertheless, the model probably performs with a margin of accuracy acceptable for health service planning at provincial and district levels, allowing for sub-national setting of priorities and resources.

The model development and results presented in this study raise several important questions that require further attention. The current lag time between data being generated (patients treated at a facility) and nationwide HMIS data being available for analysis is approximately 2 y. If predictions of treatment burden are to be made current, then the modelling framework must be extended to enable predictions at times with no contemporary data. A possible approach is to integrate the nationwide HMIS data with data from a much smaller number of “sentinel” facilities, where systems are put in place to obtain reliable data on a month-by-month basis, and to use these up-to-date data to inform the prediction from the full dataset. A second question is how many of these sentinel facility sites would be needed to achieve this purpose with an acceptable level of accuracy, and how their locations might be chosen so as to optimise their utility.

The Kenyan HMIS is typical of those found in many sub-Saharan African countries. Complex national health surveillance systems require substantial financial support and a motivated workforce within the health sector. In many resource-poor countries, ministries of health may be confronted with decisions between, say, buying drugs and printing HMIS forms. The quality of Kenya's HMIS is a symptom of an underfunded government sector. There is an urgent need to upgrade HMIS across Africa to provide reliable and timely data that are absolutely critical to planning and monitoring health service provision for disease-specific priorities [ 3, 14, 36, 37]. In the short term, we believe that the utility of even grossly incomplete HMIS data for planning national and sub-national needs can be greatly enhanced using appropriate statistical models.

## Supporting Information

Figure S1Empirical Relationship between the Size of Subsets of the Test Dataset and the Standard Deviation of Their Mean Prediction ErrorsSubsets of different sizes *n* were created from the test set by aggregating across space (by district, province, and nationally) and through time (by month and year), and the mean prediction error *μ*_ɛ_ of each subset was calculated. These subsets were then placed in bins according to their size *n,* and the standard deviation of the mean errors in each bin, σ( *μ*_ɛ_), was calculated. The *x*-axis position of each point represents the mean subset size in that bin. The theoretical relationship 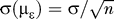 is shown (line). The purpose of the exercise was to validate the use of this equation as a model for the effect of aggregation on the variance of prediction error.
(142 KB EPS)

Protocol S1 Space–Time Kriging (27 KB DOC)

**Editors' Summary**

**Background.**

In order to allocate health-care resources (such as doctors, nurses, hospital beds, and drugs), public health officials need to know when and where in their country people are getting sick with which diseases. In most African countries, a country-wide health management information system (HMIS) compiles records about how many patients are being diagnosed with and treated for certain diseases. The actual data are meant to be collected and reported monthly by the individual health-care facilities. The HMIS compiles and analyzes these records, giving a picture of which patients are being treated across districts, regions, and the entire country. Ideally, all facilities report their data promptly and comprehensively every month. This allows the construction of a matrix that shows which treatments are used across the country through space (where) and time (when). However, many of the facilities operate under difficult circumstances, and keeping detailed records and reporting them every month is not always at the top of the priority list. As a result, data from many of the facilities are missing for any given month, and the overall national picture is inevitably incomplete.

**Why Was This Study Done?**

Almost any survey has to deal with some missing data, and there are various methods to estimate this missing data. Such estimates get harder the more data are missing. When it comes to reports on using health services in Africa, often more than half of the data are missing for a given month. Using sophisticated statistical methods instead of crude estimates is likely to make a big difference when such a big part of the data is missing. The researchers who did this study have adopted a statistical method called kriging to estimate missing data on health service usage. Kriging was originally developed in the earth sciences (such as geology and soil science) for estimating mineral concentrations at locations where no sampling had been done. This study was done to see whether kriging could be used to estimate the missing data on malaria cases in the Kenyan public health system. A better estimate of the missing data would be helpful for allocating malaria treatments to the right places.

**What Did the Researchers Do and Find?**

They obtained the monthly records of diagnoses made at outpatient departments of 2,165 health facilities across Kenya for an 84-month period from January 1996 to December 2002. The records included the number of outpatients and their diagnoses. The researchers chose to focus on malaria, for three reasons: (1) malaria is common (accounting for over one-third of the overall diagnoses in Kenya), (2) there is great variation in where and when it occurs across Kenya, and (3) donors are willing to provide additional support for malaria treatment and prevention but require documentation that such help is needed and reaches patients. The numbers of people diagnosed with malaria at each facility for a given month were matched to an independent database that contains information on where every health-care facility is located. Reporting rates varied from month to month and facility to facility, but the overall reporting rate was only 35%, with 25% of the facilities never reporting. The authors then adopted a version of kriging called space–time kriging to fill in missing data (space–time kriging assumes that for a given month a facility that didn't report is likely to be similar to its neighbors, and likely to be more similar to its own and its neighbors' recent numbers than to those further removed in space or time). The calculations resulted in a number of estimates. To test whether these estimates were accurate, the researchers randomly removed a test set of 10% of the monthly records from the full dataset and repeated the estimates based on the remaining 90% of reports. They found that the real and predicted cases across the country differed by less than 1%. At the district level (which is arguably the most useful for most planning purposes), the researchers found that their method can estimate 95% of the malaria cases within 35% of the true value. For 75% of the districts the estimates would be within 15% of the actual numbers.

**What Do These Findings Mean?**

In this case, space–time kriging provided a more precise estimate of missing data on diagnoses at the district and provincial levels than other estimates. This is likely to be true not just for malaria but for other diagnoses for which the number and the proportion of patients who have the disease and seek treatment vary by place and time of year. One caveat is that space–time kriging requires a detailed map of where exactly a country's health-care facilities are located. A database based on such a map existed for Kenya (and was used in this study) but doesn't exist in all countries that might benefit from a method like the one described here. The authors argue that knowledge about where health services are located is a must for any health planning agency, and that databases with that information should be developed everywhere.

**Additional Information.**

Please access these Web sites via the online version of this summary at http://dx.doi.org/10.1371/journal.pmed.0030271.

• Wikipedia's page on kriging (Wikipedia is a free Internet encyclopedia that anyone can edit)

• Description of, and access to, a publication entitled “Developing Health Management Information Systems: A Guide for Developing Countries” from the World Health Organization

• The Health Metrics Network, a global collaboration focused on strengthening health information systems

• The Roll Back Malaria Global Partnership, a multilateral initiative with the stated goal of halving the burden of malaria by 2010

• The Partnership in Statistics for Development in the 21st Century Description, a global initiative to promote a culture of evidence-based policymaking and monitoring

• The Kenyan Ministry of Health

## Abbreviations

HMIS: health management information system(s)
MC: malaria cases
MMTC: mean monthly total cases
SMC: standardised malaria cases
STK: space–time kriging
TC: total cases
