# Supplementary material for: Improving Imperfect Data from Health Management Information Systems in Africa Using Space–Time Geostatistics
Source: PLoS Med. 2006 Jun 6;3(6):e271. doi: 10.1371/journal.pmed.0030271 (PMC1470663; doi:10.1371/journal.pmed.0030271)
Supplement: Supplementary file 1 [file pmed.0030271.sd001.doc]

Protocol S1. Space-time Kriging

**(i) Theory**

Consider a set of data distributed in space and time, *z*(**u**_α,_*t*_α_), of an attribute *z* at *n* locations (**u**_α_, *t*_α_), α = 1,2,…, *n*, where **u** is a vector of spatial coordinates and *t* is a point in time. A space-time geostatistical problem is to predict values of *z* at a set of *m* unobserved locations, (**u***_o_*, *t_o­_*), *z**(**u** *_o_*, *t_o­_*), *o* = 1,2,…, *m*, where the asterisk denotes a prediction. The cornerstone of geostatistics is the exploitation of autocorrelation between dispersed values of *z* to make predictions at unsampled points using techniques such as kriging. Along with the data, *z*(**u**_α_, *t*_α_), space-time kriging (STK) predictors require estimates of the spatio-temporal covariance between points separated by different spatial lags, **h***_s_*, vectors of distance and direction, and temporal lags, h*_t_*, separations in time. These estimates are typically provided by first estimating the semivariance, γ, using the data at a series of regular spatial and temporal lags and fitting a continuous 2-D model to these estimates. The experimental space-time variogram is estimated by half the mean squared difference between data separated by a given spatial and temporal lag (**h***_s_*, *h_t_*):

$\hat{\gamma}_{s,t}\left(\mathbf{h}_{s},\mathrm{h}_{t}\right)=\frac{1}{2n\left(\mathbf{h}_{s},\mathrm{h}_{t}\right)}\sum_{\alpha=1}^{n\left(\mathbf{h}_{s},\mathbf{h}_{t}\right)} \left[z\left(\mathbf{u}_{\alpha},t_{\alpha}\right)-z\left(\mathbf{u}_{a}+\mathbf{h}_{s},t_{\alpha}+\mathrm{h}_{t}\right)\right]^{2}$ (1)

A suitable 2-D function is then fitted to the experimental variogram and used as input into the STK algorithm to estimate a covariance value at any given space-time lag. The STK system predicts *z**(**u**, *t*) as a linear combination of *p* data proximate in space and time:

$z*(\mathbf{u},t)=\sum_{\alpha=1}^{p(\mathbf{u},t)} \lambda_{\alpha}(\mathbf{u},t)z(\mathbf{u}_{\alpha},t_{\alpha})$ (2)

The utility of kriging approaches lies in the ability to determine the weight, *λ_α_*(**u**, *t*), assigned to each neighbouring datum in order to minimise the prediction variance, $\sigma_{E}^{2}(\mathbf{u},t)$:

$\sigma_{E}^{2}(\mathbf{u},t)=Var[z*(\mathbf{u},t)-z(\mathbf{u},t)]$ (3)

whilst maintaining unbiasedness of the estimator *z**(**u**, *t*). In determining optimum weights, kriging takes into account both the covariance between each datum and the point to be estimated, and the covariances between data themselves.

**(ii) Implementation**

The separate STK exercises to predict *TC and *SMC were composed of the following methodological steps.

(i) Data were separated into three facility classes (hospitals, health centres and dispensaries) and all analysis was carried out separately for each facility class.

(ii) The space-time experimental variogram was estimated for each facility class (Eq. 1). This procedure was carried out using the modified GSLIB routine [25] provided by De Cesare *et al*. [27]. Variograms were modelled up to spatial lags of 100 km and temporal lags of 24 months. Since the objective was to interpolate (fill in gaps), rather than to extrapolate (predict into the future), time was considered isotropic (i.e. temporal lag was defined only by the number of months, and not by direction in time).

(iii) A general product-sum space-time variogram model was fitted to each sample variogram surface [26]. This class of model was chosen since it guarantees invertability of the space-time covariance matrix (a fundamental step in the kriging system) and allows a straightforward method for model fitting. The necessary parameters of the product-sum model can be estimated by fitting a conventional 2-D variogram model, composed of the usual suite of valid models (see [25] p. 25) to each of the space- and time-marginal sample variograms, and by estimation of the space-time sill, which models semivariance at spatial and temporal lags beyond the range of spatio-temporal autocorrelation. The separate space-and time-marginal variograms were obtained from the space-time sample variogram by setting *h_t_* = 0 and **h***_s_* = 0, respectively.

(iv) Parameters for each space-time variogram model were input into the STK algorithm [27] along with the corresponding TC or SMC data for each facility class and predictions were made for all missing records.
